# Pattern of seat belt use by drivers in Trinidad and Tobago, West Indies

**DOI:** 10.1186/1756-0500-4-201

**Published:** 2011-06-16

**Authors:** Abiodun Olukoga, George Legall, Abayomi Odekunle

**Affiliations:** 1 Department of Para-Clinical Sciences, Faculty of Medical Sciences, University of the West Indies, St. Augustine, Trinidad, WI; 2 Department of Pre-Clinical Sciences, Faculty of Medical Sciences, University of the West Indies, St. Augustine, Trinidad, WI

## Abstract

**Background:**

In Trinidad and Tobago, the law on the mandatory use of seat belts was passed in 1995, but this law is hardly enforced. The objective of this study was to determine the frequency and predictors of seat belt use by motor vehicle drivers in the country.

**Findings:**

A cross-sectional study of 959 motor vehicle drivers using a self-administered questionnaire. Data analysis included Pearson Chi square test and multinomial logistic regression analysis in order to determine the possible predictors of seat belt use by the drivers in Trinidad and Tobago. A majority of the drivers sometimes (51.8%) or always (31.6%) use a seat belt. About 16.7%, 29% and 54.2% of the drivers perceived that the other drivers use their seat belts more frequently, with the same frequency and less frequently respectively compared to themselves. The main reason for not using seat belt by the drivers was given as frequent stops (40.7%) and the main motivation to use seat belt by the drivers was given as stiffer penalties for non-compliance with the seat belt law (44.5%). The predictors of seat belt use were male driver, no formal or lower level of education, driving for less than 10 years, and the perception that the other drivers use seat belts with the same or higher frequency compared to the respondents.

**Conclusion:**

Only a small proportion of the drivers in Trinidad and Tobago always use a seat belt when driving. There is the need to enforce the seat belt legislation in the country.

## Findings

Road traffic accidents constitute a public health challenge worldwide [1,2]. In Trinidad and Tobago, there has been an increase in the number of road traffic accidents in the country, with the associated injuries and fatalities. In many of these road traffic accidents, most of the drivers and passengers involved as casualties and fatalities were reported as not using seat belts while the vehicles were moving [3,4].

Motor vehicle seat belts have been shown to be cost-effective protective devices that reduce the risk and severity of injuries and the risk of death in road traffic accidents [5-8]. The use of seat belts by the occupants of motor vehicles has been shown to result in significant reductions in the number and severity of injuries, and the occurrence of fatalities in the event that a road traffic accident occurs [9][5]. Studies have shown that the use of seat belts results in the reduction of drivers and passengers fatalities by 45% and reduction of serious injuries to the head, chest and extremities by more than 50% [10]. If the occupants of a motor vehicle that is involved in a road traffic accident are using their seat belts properly, they are retained in their seats and this prevents them from hitting objects around them and being propelled or ejected out of the vehicles through the windscreens or windows [6].

In Trinidad and Tobago, the law on the mandatory use of seat belts was passed in 1995 [4]. But, this law is applicable to only the drivers and front seat passengers. The law stipulates a fine of TT$500 (about US$80) for the contravention of this seat belt law. However, this law is not enforced in anyway and it would be strange to find anyone charged for this traffic offence in the country. The objective of this study was to determine the frequency of seat belt use by motor vehicle drivers in Trinidad and Tobago and the factors that influence seat belt use.

This was a cross-sectional study designed to determine the frequency of seat belt use by motor vehicle drivers in Trinidad and Tobago and the factors that influence seat belt use. The participants were 1150 motor vehicle drivers in selected places across Trinidad and Tobago. The locations for the survey were chosen in order to give a nationally representative sample. Participation was voluntary and all responses were anonymous.

Informed consent was obtained verbally from the participants after the purpose of the study was explained to them. In addition, each questionnaire had an information sheet attached with it.

Each of the participants completed a self-administered questionnaire that had questions on the drivers' frequency of seatbelt use, knowledge of seat belt regulations, awareness of penalty for seat belt non-use when driving and basic socio-demographic variables. The questionnaire consisted mostly of closed-ended questions and allowed for only single responses by the participating drivers. The Ethics Committee, Faculty of Medical Sciences, the University of the West Indies, St. Augustine, Trinidad, West Indies granted ethical approval for this study.

For the purposes of data analysis, the participants were categorized into three groups i.e. never use seat belts, sometimes use seat belts and always use seat belts when driving. Pearson Chi square test was done to test for statistically significant association between the socio-demographic variables, selected questions on seat belt use and the three different categories of seat belt use by the participating drivers. A multinomial logistic regression analysis was done in order to determine the possible predictors of seat belt use by the participating drivers.

## Results

A total of 1150 questionnaires were returned out of the 1500 that were administered giving a response rate of 76.7% by the participating drivers. Some of the returned questionnaires were discarded from the analysis due to incomplete data. Only 959 usable questionnaires with valid and sufficient responses were analysed.

### Basic demographics

Table 1 shows the basic demographics of the 959 responding drivers. The drivers use seat belt sometimes (51.8%), always (31.6%) and never (16.6%) when driving.

**Table 1:** Demographics and frequency of seat belt use

Variable	Never (%)		Sometimes (%)		Always (%)		Total (%)		Chi square	P value
Age (years)										
18-24	33	(20.8)	138	(27.8)	70	(23.1)	241	(25.1)	27.480	< 0.001
25-34	69	(43.4)	161	(32.4)	75	(24.8)	305	(31.8)		
35-44	22	(13.8)	102	(20.5)	68	(22.4)	192	(20.0)		
>/45	35	(22.0)	96	(19.3)	90	(29.7)	221	(23.0)		
Total	159	(100.0)	497	(100.0)	303	(100.0)	959	(100.0)		
Sex										
Male	144	(91.7)	366	(75.6)	141	(49.6)	651	(70.4)	99.223	< 0.001
Female	13	(8.3)	118	(24.4)	143	(50.4)	274	(29.6)		
Total	157	(100.0)	484	(100.0)	284	(100.0)	925	(100.0)		
Marital Status										
Single	90	(57.3)	271	(56.0)	127	(44.6)	488	(52.7)	11.229	.024
Married	57	(36.3)	186	(38.4)	137	(48.1)	380	(41.0)		
Divorced/Widowed	10	(6.4)	27	(5.6)	21	(7.4)	58	(6.3)		
Total	157	(100.0)	484	(100.0)	285	(100.0)	926	(100.0)		
Education										
No formal education	2	(1.3)	16	(3.3)	1	(.3)	19	(2.1)	169.350	< 0.001
Primary	43	(27.4)	47	(9.8)	4	(1.4)	94	(10.2)		
Secondary	93	(59.2)	241	(50.00	94	(32.8)	428	(46.2)		
Tertiary	19	(12.1)	178	(36.9)	188	(65.5)	385	(41.6)		
Total	157	(100.0)	482	(100.0)	287	(100.0)	926	(100.0)		
Years driving										
0 - 9	87	(54.7)	233	(46.9)	99	(32.7)	419	(43.7)	26.913	< 0.001
10 - 19	33	(20.8)	132	(26.6)	89	(29.4)	254	(26.5)		
>/20	39	(24.5)	132	(26.6)	115	(38.0)	286	(29.8)		
Total	159	(100.0)	497	(100.0)	303	(100.0)	959	(100.0)		

There were more male drivers (70%) compared to female drivers (30%). About one-half of the drivers were single and married drivers were 41%. Most of the drivers (88%) have at least secondary education. Most of the drivers have been driving for less than 10 years (43.7%). There was a significant difference in the use of seat belts by the drivers with respect to all the socio-demographic variables i.e. age (x^2 ^= 27.480, p < 0.005), sex (x^2 ^= 99.223, p < 0.005), marital status (x^2 ^= 11.229, p < 0.05), education (x^2 ^= 169.350, p < 0.005) and years of driving (x^2 ^= 26.913, p < 0.005).

### Responses to selected questions on seat belt use

Table 2 shows the responses of the drivers to some selected questions on seat belt use by the pattern of seat belt use by the drivers. A vast majority of the drivers (96.6%) in this study had functional seat belts in their vehicles. An equally vast majority of the drivers (96.1%) were aware of the seat belt law in the country. There was a high level of awareness (87.8%) of the fine for the non-use of seat belt amongst the interviewed drivers.

**Table 2:** Responses to selected questions on seat belt use

Variable	Never (%)		Sometimes (%)		Always (%)		Total (%)		Chi square	P value
Driver's seat of vehicle has a seat belt										
No	12	(7.5)	19	(3.8)	2	(.7)	33	(3.4)		
Yes	147	(92.5)	478	(96.2)	301	(99.3)	926	(96.6)	15.339	< 0.001
Total	159	(100.0)	497	(100.0)	303	(100.0)	959	(100.0)		
Knowledge of seat belt law in Trinidad & Tobago										
No	15	(9.4)	12	(2.4)	10	(3.3)	37	(3.9)		
Yes	144	(90.6)	485	(97.6)	293	(96.7)	922	(96.1)	16.373	< 0.001
Total	159	(100.0)	497	(100.0)	303	(100.0)	959	(100.0)		
Awareness of penalty for seat belt non- use										
No	25	(15.7)	66	(13.3)	26	(8.6)	117	(12.2)		
Yes	134	(84.3)	431	(86.7)	277	(91.4)	842	(87.8)	6.088	.048
Total	159	(100.0)	497	(100.0)	303	(100.0)	959	(100.0)		
Respondents use of seat belts compared to other drivers										
The same	46	(39.0)	104	(26.8)	71	(28.0)	221	(29.1)	75.243	< 0.001
More than I do	39	(33.1)	78	(20.1)	10	(3.9)	127	(16.7)		
Less than I do	33	(28.0)	206	(53.1)	173	(68.1)	412	(54.2)		
Total	118	(100.0)	388	(100.0)	254	(100.0)	760	(100.0)		
Main reason for not using seat belt										
Discomfort	25	(15.7)	116	(23.3)	0		141	(21.4)	59.894	< 0.001
Forgetfulness	6	(3.8)	97	(19.5)	1	(33.3)	104	(15.8)		
Habit	27	(17.0)	74	(14.9)	0		101	(15.3)		
Frequent stops	94	(59.1)	174	(35.0)	0		268	(40.7)		
Others	7	(4.4)	36	(7.2)	2	(66.7)	45	(6.8)		
Total	159	(100.0)	497	(100.0)	3	(100.0)	659	(100.0)		
Motivators for drivers to use seat belts										
Increased mass media promotion	53	(34.0)	161	(32.8)	128	(43.8)	342	(36.4)	11.558	.021
Stiffer penalties for non-compliance	71	(45.5)	226	(46.00	121	(41.4)	418	(44.5)		
Increased traffic police patrols	32	(20.5)	104	(21.2)	43	(14.7)	179	(19.1)		
Total	156	(100.0)	491	(100.0)	292	(100.0)	939	(100.0)		

• There is a reporting bias in the reports of seat belt availability and usage by the 33 drivers who do not have seat belts in their vehicles. These 33 drivers had responded that they never (12), sometimes (19) and always (2) use seat belts.

The drivers were requested to compare their use of seat belts to that of the other drivers. Interestingly, only 16.7% of the drivers perceived that the other drivers use their seat belts more frequently than they do and 54.2% perceived that the other drivers use their seat belts less frequently compared to them.

The reasons for not using seat belt by the drivers were given as frequent stops (40.7%), discomfort (21.4%), forgetfulness (15.8%) and habit (15.3%). The motivations to use seat belt by the drivers were given as stiffer penalties for non-compliance with the seat belt law (44.5%), increased mass media promotion (36.4%) and increased traffic police patrols (19.1%).

There was a significant difference in each of the 6 responses of the drivers to the selected questions on seat belt use and the pattern of seat belt use by the drivers use in terms of possession of seat belts in their vehicles (x^2 ^= 15.339, p < 0.005), knowledge of the seat belt law in the country (x^2 ^= 16.373, p < 0.005), awareness of the penalty for not using seat belt (x^2 ^= 6.088, p < 0.05), comparison of the respondents use of seat belts to that by the other drivers (x^2 ^= 75.243, p < 0.005), main reason for not using seat belt (x^2 ^= 59.894, p < 0.005) and the motivators for drivers to use seat belts (x^2 ^= 11.558, p < 0.05).

### Multinomial logistic regression model for predicting the determinants of seat belt use

Table 3 shows the result from the multinomial logistic regression model for the three categories of use seat belt (never use seat belt is the reference group). The table presents the logistic regression coefficients and odds ratios for the independent variables as they relate to the driver sometimes using seat belt and always using seat belt while driving.

**Table 3:** Multinomial logistic regression model for seat belt use

	Sometimes				Always			
**Parameter**	**Odds Ratio**	**95% CI**		**p**	**Odds Ratio**	**95% CI**		**p**

Age (years)								
18-24	1.980	.645	6.073	.233	1.345	.354	5.101	.663
25-34	.769	.286	2.067	.603	.460	.146	1.456	.187
35-44	1.191	.522	2.715	.678	.892	.353	2.251	.808
Sex								
Male	.251	.094	.668	.006	.146	.053	.402	< 0.001
Marital status								
Single	.557	.175	1.771	.322	.411	.112	1.503	.179
Married	.460	.149	1.418	.176	.433	.124	1.508	.189
Education								
No formal education	1.051	.195	5.666	.954	.067	.005	.932	.044
Primary	.115	.052	.257	< 0.001	.012	.003	.049	< 0.001
Secondary	.278	.138	.560	< 0.001	.166	.078	.353	< 0.001
Years driving								
0 - 9 years	.547	.208	1.438	.221	.304	.097	.947	.040
10 - 19 years	1.133	.491	2.616	.770	.891	.347	2.289	.811
Perception								
The same	.449	.257	.784	.005	.324	.173	.608	< 0.001
More than I do	.369	.205	.663	.001	.048	.019	.122	< 0.001
Motivation								
Increased mass media promotion	.850	.434	1.663	.635	1.751	.778	3.944	.176
Stiffer penalties for non-compliance	.889	.464	1.702	.722	1.437	.646	3.197	.375

• Reference categories were Age: >/45 years, Sex: Female; Marital status; Divorced/widowed; Education: Tertiary education; Years driving: >/20 years

The variables that predicted if the driver was less likely to sometimes use seat belts were being a male driver, lower level of education, perception by the driver that the other drivers use seat belts with the same or higher frequency compared to themselves. The variables that predicted if the driver was less likely to always use seat belts were being a male driver, no formal or lower level of education, driving for less than 10 years, and the perception by the driver that the other drivers use seat belts with the same or higher frequency compared to them.

## Discussion

This study analysed the frequency of seat belt use, the knowledge and awareness of aspects of the seat belt regulation, and the influence of the socio-demographic variables on seat belt use by motor vehicle drivers in Trinidad and Tobago.

The proportion of drivers (96.6%) with seat belts in their vehicles in this study is consistent with the figures reported for drivers in Kingston, Jamaica (99.9%) and two Nigerian cities of Benin (89%) and Ibadan (95%) [12-14]. The few drivers (3.4%) without functional seat belts in their vehicles are in contravention of the road traffic laws of the country. According to the laws of Trinidad and Tobago (Chapter 48:50 of the Motor Vehicle and Road Traffic Act), it is mandatory for every vehicle to be fitted with seat belts for the driver and front seat passengers, irrespective of whether the vehicle was imported, locally manufactured or assembled [11].

About 83% of the drivers sometimes or always use a seat belt while driving. This is similar to the 81% reported for private motor vehicle drivers in Kingston, Jamaica in 2004; 91% for the mini-bus drivers, in Osogbo, Nigeria and 88% for the commercial motor vehicle drivers in Hawaii [12], [15,16].

The proportion of drivers who always use their seat belts (31.6%) in Trinidad and Tobago is low compared to the drivers in Osogbo, Nigeria (47%) and Hawaii (67%) respectively [15,16].

The Motor Vehicle and Road Traffic Act of Trinidad and Tobago, makes it mandatory for the driver and front seat passengers to use seat belts when the vehicle is in motion. But as mentioned in the Introduction, this and other related laws are rarely enforced. Police officers often cite the endless bureaucracy associated with prosecution of offending motorists in the country's courts as a hindrance in enforcing the seat belt laws [4].

The low proportion of drivers who always use seat belt in Trinidad and Tobago is particularly disturbing when it is taken into account that the results of this study were based on self-reports. This is because the use of self-reports has been shown to result in higher rates of seat belt use compared to direct observation [17]. Hence, it could be concluded that the proportion of drivers who always use seat belt in Trinidad and Tobago would be much lower than the 31.6% obtained in this study in a similar study using direct observation rather than self-report.

Reported seat belt usage rates vary depending on the country. Countries with reported low seat belt use rate for drivers include Lebanon (12%), Turkey (21%), Thailand (31%) and Kuwait (50%). In Saudi Arabia, the seat belt use rates for drivers were 33% and 87% respectively in two Riyadh districts [6]. But, in most industrialised countries much higher seat belt usage rates have been reported, such as 70% in urban roads in Norway in 1995, about 81% in the USA in 2006, 81% in Victoria (Australia) in 1994 and 90% in the UK in 1983 after making seat belt use compulsory [9].

The 96% level of awareness of the seat belt law in Trinidad and Tobago was higher than the 85% level of awareness of the seat belt law by the commercial motor vehicle drivers in Hawaii [16]. Most (88%) of the drivers were aware of a fine for not using seat belt. It is interesting that the level of awareness of the seat belt law (96%) was slightly higher than the level of awareness of the fine for non-use of seat belt (88%). This simply means that there are drivers who know about the seat belt law but are unaware of the fine for not using their seat belts when driving.

The proportion of the drivers (88%) in this study who were aware of a fine for not using seat belt compares favourably with the 95% of drivers in Osogbo, Nigeria and 74% of the commercial motor vehicle drivers in Hawaii who were aware of the imposition of a fine when they do not use their seat belts while driving [15,16]. In this study, the drivers were not asked to state the fine for not wearing a seat belt. But, only 8 out of 572 i.e. 1.4% of the commercial motor vehicle drivers in Hawaii knew the correct fine (US$92) for not wearing a seat belt [16].

The majority of the drivers (41%) who were aware of the fine for the non-use of seat belt have been driving for less than 10 years. This is similar to the proportion of drivers (49%) who aware of the fine for the non-use of seat belt in Osogbo, Nigeria [15].

The drivers in this study perceive themselves as above average in their use of seat belts when compared with other drivers in the country. They rated themselves as using their seat belts more frequently than 54%, with the same frequency as 29% and less frequently as 17%, of the other drivers. In a similar vein, the commercial motor vehicle drivers in Hawaii rated themselves as better than the other drivers who they perceived as always (31%), sometimes (51%) and never (18%) use a seat belt while driving. But, these Hawaiian drivers reported that they always (67%), sometimes (21%) and never (11%) use a seat belt while driving [16].

Our study showed that some variables predict whether a driver was less likely to sometimes or always use seat belts when driving. These variables were being a male driver, no formal or lower level of education, driving for less than 10 years, and the perception by the driver that the other drivers use seat belts with the same or higher frequency compared to them.

We found that age, marital status and the drivers' perceived motivators for seat belt use did not predict whether a driver sometimes or always use seat belts. The findings that the age of the driver did not predict seat belt use are consistent with the findings by Fernandez et. al. (2006) in Boston, Massachusetts [10]. However, we found that the level of education of the driver is a predictor of seat belt use. This is at variance with the findings by Fernandez et. al. (2006) who found no such association between seat belt use and the level of education of the driver [10]. This difference could be due to the fact that the study by Fernandez et. al. (2006) was conducted amongst patients in the emergency department of a hospital unlike our study which was conducted amongst the general population.

Overall, our results suggest that in Trinidad and Tobago, a low proportion of the drivers self-reported always using seat belt when driving. Based on these results, concerted efforts are needed to increase seat belt use in the driving population in the country.

## Competing interests

The authors declare that they have no competing interests.

## Authors' contributions

All authors participated in the design of the study and the discussion of findings. AO^1 ^and GL executed the data management, AO^1 ^drafted the manuscript, AO^1 ^and GL revised the manuscript. All authors read and approved the final manuscript.
